# Identification of a Novel Mutation in the SERPINE1 Gene Causing Clinical Hyperfibrinolysis in English Springer Spaniel Dogs

**DOI:** 10.1111/jvim.70150

**Published:** 2025-06-05

**Authors:** Kelley Kilpatrick, Jonah N. Cullen, Farah F. Almeer, LeeAnn Higgins, Todd Markowski, Marjory Brooks, Steven G. Friedenberg, Molly Racette

**Affiliations:** ^1^ Department of Medical Sciences, School of Veterinary Medicine University of Wisconsin Madison Wisconsin USA; ^2^ Department of Veterinary Clinical Sciences, College of Veterinary Medicine University of Minnesota St. Paul Minnesota USA; ^3^ Department of Biochemistry, Molecular Biology, and Biophysics, College of Biological Sciences University of Minnesota Minneapolis Minnesota USA; ^4^ Department of Population Medicine and Diagnostic Sciences, College of Veterinary Medicine Cornell University Ithaca New York USA

**Keywords:** coagulation, delayed postoperative hemorrhage, plasminogen activator inhibitor‐1, whole genome sequencing

## Abstract

**Background:**

A 7‐month‐old female spayed English Springer Spaniel (ESS) was evaluated for spontaneous hemoperitoneum. Hyperfibrinolysis was identified on thromboelastography.

**Hypothesis/Objectives:**

To identify a genetic mutation causing congenital hyperfibrinolysis in the proband and evaluate the prevalence of the mutation in the ESS breed.

**Animals:**

Client‐owned ESS with hemorrhage and a non‐affected littermate. Samples of DNA from 3 ESS, 1 Welsh Springer Spaniel (WSS) with unexplained hemorrhage, and 199 ESS with no history of hemorrhage.

**Methods:**

Whole genome sequencing (WGS) of the proband with variant filtering against an in‐house WGS database of 671 presumably unaffected dogs identified a deleterious variant of *SERPINE1* unique to the proband, which encodes for plasminogen activator inhibitor 1 (PAI‐1). *SERPINE1* was genotyped in the remaining animal population by Sanger sequencing or a Taqman assay. Liquid chromatography tandem mass spectrometry (LC–MS/MS) was performed on platelet pellets from the proband, a littermate, and three unrelated healthy ESS.

**Results:**

Whole genome sequencing of the proband identified a unique homozygous insertion at chr6:8640592 in exon 1 of *SERPINE1*, which is predicted to cause a premature stop codon. The unaffected littermate was heterozygous for the mutation. Two unrelated ESS and 1 WSS with post‐operative hemorrhage were homozygous for the mutation. Absence of PAI‐1 in the proband's platelets was documented using LC–MS/MS.

**Conclusions and Clinical Importance:**

This novel mutation in *SERPINE1* is associated with the absence of the PAI‐1 protein in platelets and might cause hemorrhage because of hyperfibrinolysis in ESS and related breeds.

**Abbreviations**
BMBT: buccal mucosal bleeding time
DEPOH: delayed postoperative hemorrhage
ESS: English Springer Spaniel
LC–MS/MS: liquid chromatography–tandem mass spectrometry
PAI‐1: plasminogen activator inhibitor‐1
PSM: peptide‐spectrum match
PT: prothrombin time
PTT: partial thromboplastin time
TCT: thrombin clotting time
TEG: thromboelastography
tPA: tissue plasminogen activator
uPA: urokinase
WGS: whole genome sequencing
WSS: Welsh Springer Spaniel

## 1 Introduction

Primary hyperfibrinolysis is caused by dysfunction of proteins in the fibrinolytic pathway, which leads to accelerated clot breakdown [1]. Hastened clot dissolution can lead to hemorrhage that manifests as cutaneous, subcutaneous, or cavitary bleeding. Primary hyperfibrinolysis can occur because of an increase in activators of fibrinolysis (e.g., tissue plasminogen activator [tPA], plasminogen, plasmin) or a decrease in the activity of inhibitors of fibrinolysis [1]. Inhibitors of the fibrinolytic system include plasminogen activator inhibitor‐1 (PAI‐1), alpha‐2 antiplasmin, and thrombin activatable fibrinolysis inhibitor (TAFI) [2]. Plasminogen activator inhibitor‐1, the main inhibitor of fibrinolysis, inactivates both tPA and urokinase (uPA) by creating complexes with plasminogen that impair fibrinolysis [3].

In humans, congenital PAI‐1 deficiency has been described as a cause of bleeding diatheses associated with trauma [4], menstruation [4, 5, 6, 7], pregnancy [8, 9], and surgery [10, 11, 12, 13]. In animals, to the best of our knowledge, congenital PAI‐1 deficiencies have not been described previously. However, significantly lower alpha‐2 antiplasmin concentrations have been documented in a case series of Greyhounds with abnormal post‐operative bleeding compared with “non‐bleeder” control Greyhounds [14]. A genome‐wide association study of Scottish Deerhounds with and without delayed post‐operative hemorrhage (DEPOH) identified a missense variant in *SERPINF2*, the gene encoding alpha‐2 antiplasmin, associated with the clinical bleeding phenotype [15].

Our purpose is to describe an investigation into the cause of unexplained DEPOH in a young English Springer Spaniel (ESS), as well as its implications for the breed more broadly. The clinical features, results of whole genome sequencing (WGS), and follow‐up sequencing of variants in a littermate and unrelated English and Welsh Springer Spaniels (WSS) with delayed and recurrent post‐operative hemorrhage are reported.

## 2 Materials and Methods

### 2.1 Animal Selection and Phenotyping

The proband, a 7‐month‐old female spayed ESS, was referred to the Emergency Service at the University of Wisconsin Veterinary Care for evaluation of recurrent subcutaneous and peritoneal hemorrhage episodes 6 weeks after routine ovariohysterectomy. Evaluation of the proband included physical examinations, CBCs, serum biochemistry panels, coagulation testing, abdominal computed tomography (CT), and an exploratory laparotomy. The proband had been tail docked by the breeder, but the current owner was not informed of bleeding concerns at the time of tail docking.

The owner of one of the proband's littermates, who reported that the dog did not have clinical evidence of hemorrhage after castration, was contacted and agreed to enroll the dog in our study. This dog was also tail‐docked by the breeder with no bleeding concerns reported to the current owner. Owners of the proband's parents and the other two littermates could not be reached.

Archival DNA samples from the Cornell University Veterinary Biobank were analyzed from 3 ESSs and 1 WSS with histories of delayed and prolonged post‐operative hemorrhage of unknown cause, and from 196 ESSs with no history of unexplained hemorrhage collected for an epilepsy study between 1999 and 2001 at the University of Minnesota Canine Genetics Laboratory. These latter ESS dogs ranged in age from 6 months to 15 years and included 62 males, 89 females, and 45 dogs for which sex was unavailable.

Three additional healthy, unrelated ESS were enrolled in the study from the University of Wisconsin to collect DNA and platelet samples for genetics and proteomics analyses: a 3‐year‐old female spayed, a 2‐year‐old female spayed, and a 2‐year‐old male castrated dog.

### 2.2 Sample Preparation

Approximately 3 mL of ethylene diamine‐tetra acetic acid (EDTA)‐anticoagulated blood was collected from the proband, a littermate, and three unrelated ESS and shipped at room temperature to the Canine Genetics Laboratory at the University of Minnesota College of Veterinary Medicine (UMN‐CVM). Samples of DNA were extracted using a Puregene kit (Qiagen, Germantown, MD) following the manufacturer's recommended protocol. Platelet concentrates from the proband, a littermate, and three additional healthy ESS were prepared from 10 mL acid citrate dextrose‐A anticoagulated whole blood as previously described [16] with minor adjustment: the final platelet pellet was not combined with the lysis buffer and instead was frozen at −80°C. Platelet pellets were shipped on dry ice to the Center for Metabolomics and Proteomics at the University of Minnesota for further analysis. All blood sampling was performed with informed owner consent following an approved Institutional Animal Care and Use Committee (IACUC) protocol from the University of Wisconsin (protocol #V006684).

### 2.3 Genetic Analyses

Whole genome sequencing (WGS) of the proband was performed at Azenta Life Sciences (Burlington, MA). A DNA library was prepared using an Illumina TruSeq PCR‐Free Kit, and 150 base‐pair, paired‐end reads were generated on an Illumina NovaSeq 6000 system. Reads were mapped against the dog reference genome assembly UU_Cfam_GSD_1.0 [10, 11] and processed using the OnlyWAG pipeline as described [17]. Raw sequence reads are publicly available at National Center for Biotechnology Information's Short Read Archive (SRR31749359) under BioProject PRJNA937381. As part of the OnlyWAG pipeline, variants in the proband were compared to an internal database of whole genome sequences from 671 dogs of 63 diverse breeds, which did not include any ESS. Whole genome sequence data from these 671 dogs were processed using the OneWAG and ManyWAGS pipelines [17]. Variants unique to the proband were prioritized for further evaluation by expected impact as high (e.g., frame shift, loss or gain of stop codon, affecting a splice site junction), moderate (e.g., nonsynonymous point mutation), low (e.g., synonymous point mutation), or modifier (e.g., intronic, intergenic) as defined by Variant Effect Predictor [18]. High‐ and moderate‐impact variants were compared to 12 genes that have been identified previously in congenital hyperfibrinolysis in humans and dogs [19, 20, 21, 22, 23, 24, 25] (Table S1). A high‐impact variant in the *SERPINE1* gene subsequently was compared to a database of WGS from 1971 dogs released by the Dog10K consortium, which includes three ESS [26].

Based upon our WGS findings, the high‐priority variant in *SERPINE1* was genotyped by Sanger sequencing in the proband, the proband's littermate, three healthy ESS from the University of Wisconsin, and three ESS and one WSS from the Cornell Veterinary Biobank. Genomic DNA (approximately 50 ng) from each of these dogs was amplified by PCR using HotStarTaq Master Mix (Qiagen), forward primer 5′‐CGAGAAGATGGGATGAGTGC, and reverse primer 5′‐GCCATCTCCCCTCCCCATT. The PCR was performed on a BioRad (Hercules, CA) T100 thermal cycler with the following cycling conditions: hold at 95°C for 15 min, denature at 94°C for 1 min, anneal at 55°C for 1 min, extend at 72°C for 1 min. Cycling was repeated 35 times with the last extension step lasting 10 min. The PCR products were visualized by gel electrophoresis to confirm fragment length. The resulting amplicons were cleaned using ExoSAP‐IT (ThermoFisher, Waltham, MA) following the manufacturer's recommendations and sequenced by Sanger sequencing at Eurofins Genomics (Louisville, KY). Sequence files were aligned to UU_Cfam_GSD_1.0 [10, 11] and visualized using Geneious software (Biomatters, Auckland, NZ).

Samples of DNA from an additional 196 ESS with no history of clinical bleeding were genotyped using a custom proprietary TaqMan assay (ThermoFisher) containing HEX‐ and FAM‐labeled probes using the iTaq Universal Supermix for Probes (BioRad) and 30 ng DNA per sample. TaqMan genotyping was performed on a CFX96 real‐time thermal cycler (BioRad) with the following cycling conditions: hold at 95°C for 3 min, denature at 95°C for 5 s, anneal at 60°C for 30 s. Cycling was repeated 34 times. Data analysis was performed using CFX Maestro Software (BioRad).

### 2.4 Liquid Chromatography–Tandem Mass Spectrometry

Liquid chromatography–tandem mass spectrometry (LC–MS/MS) was performed on platelet concentrates from the proband in duplicate, a littermate, and three unrelated clinically healthy ESS. Platelets were chosen for this assay because of their high concentration of endogenous PAI‐1 in healthy animals [27, 28].

Protein from platelet samples was extracted with lysis buffer (7 M urea, 2 M thiourea, 0.4 Tris pH 8, 20% acetonitrile, 10 mM tris [2‐carboxyethyl] phosphine [TCEP], 40 mM chloroacetamide, and 1X HALT protease inhibitor [ThermoFisher]) and briefly vortexed, followed by sonication at 30% amplitude for 7 s using a Branson Digital Sonifier 250 (Branson Ultrasonics, Danbury, CT). Each sample was transferred to a pressure‐cycling tube for the Barocycler NEP2320 (Pressure Biosciences Inc., South Easton, MA) and cycled between 35 kPSI for 20 s and 0 kPSI for 10 s for 60 cycles at 37°C. Samples were centrifuged at 15000 *g* for 10 min, and supernatants were transferred to a new microfuge Eppendorf Protein LoBind tube. Aliquots for each sample were used for protein concentration determination by Bradford assay.

Platelet proteins were digested by transferring a 5 μg aliquot to a new microfuge tube, standardizing the volumes across samples with lysis buffer, diluting each sample five‐fold with water, and adding trypsin (Promega, Madison, WI) in a 1:40 ratio of trypsin to total protein. Samples were incubated overnight for 16 h at 37°C, acidified with 0.2% formic acid, and cleaned with an MCX‐like SDB‐RPS STAGE tip [29]. Eluates were dried in a vacuum concentrator.

Approximately 100 ng of each peptide mixture dissolved in 98:2 water: acetonitrile and 1% formic acid were analyzed by capillary LC–MS/MS on a Dionex UltiMate 3000 RSLCnano system (ThermoFisher) online with an Orbitrap Eclipse mass spectrometer (ThermoFisher) in data‐dependent acquisition mode. We performed gradient separation on a self‐packed C18 column (Dr. Maisch GmbH ReproSil‐PUR 1.9 μm 120 Å C18aq, 100 μm ID × 35 cm length) at 55°C with solvents A (0.1% formic acid in water) and B (0.1% formic acid in acetonitrile). We used the following elution profile: 5% B solvent from 0 to 2 min, 8% B at 2.5 min, 21% B at 40 min, 35% B at 60 min, and 90% B at 62 min with a flow rate of 350 nL/min from 0 to 2 min and 315 nL/min from 2.5 to 60 min. We employed the following mass spectrometry (MS) parameters: ESI voltage +2.1 kV, ion transfer tube 275°C; no internal calibration; Orbitrap MS1 scan 60 k resolution in profile mode from 380 to 1410 m/z with 50 msec injection time; 100% (4 × 10^5^) automatic gain control (AGC); MS2 was triggered during a 3 s loop cycle with a loop count of 20 for the *SERPINE1* peptide precursor values in a targeted inclusion list (Table S2). The MS2 settings were: high energy collisional dissociation (HCD); 1.2 Da quadrupole isolation window, 30% fixed collision energy, monoisotopic peak determination (MIPS) was set to Peptide; Orbitrap detection with 30 K resolution at 200 m/z, 54 msec maximum injection time and 100% (1 × 10^4^) AGC.

Peptide MS data were processed using SEQUEST (ThermoFisher) in Proteome Discoverer 3.1 [30]. The human Universal Proteome database (UP000805418) was downloaded from UniProt on 8/18/2023 and merged with a common laboratory contaminant protein database (https://blogs.gwu.edu/haolab/resources/; 43 994 total protein sequences). We applied the precursor mass recalibration node with precursor mass tolerance 20 ppm, product ion tolerance 0.08 Da with fixed carbamidomethyl (CAM) modification of cysteine 57.0215 m/z. Database search parameters were: enzyme trypsin full specificity, one missed cleave site, precursor tolerance 15 ppm, fragment ion tolerance 0.06 Da. We specified CAM cysteine (+57.021 Da) as a fixed modification and the dynamic modification was deamidation of asparagine (+0.984 Da). We applied 1% protein and peptide false discovery rate (FDR) filters using the Percolator algorithm [31] in Proteome Discoverer to identify proteins with high confidence.

## 3 Results

### 3.1 Animal Phenotyping

A 7‐month‐old female spayed ESS, weighing 17.5 kg, was presented to an emergency clinic for evaluation of lethargy 6 weeks after ovariohysterectomy. Physical examination identified a heart rate of 138 beats per minute, a holosystolic murmur, pale mucous membranes, capillary refill time of 4 s, and a fluid wave on abdominal palpation. A regenerative anemia (hematocrit, 16.2%; reference interval [RI], 37.3%–61.7%; reticulocyte count, 143.6 x 10^3^/μL; RI, 10.0–110.0 x 10^3^/μL) was identified on CBC. History confirmed no exposure to rodenticides or trauma. Point‐of‐care ultrasonography and abdominocentesis confirmed spontaneous hemoperitoneum with a packed cell volume (PCV) and total solids (TS) of 16% and 4.2 g/dL, respectively. Initial treatment included transfusion of typed, packed red blood cells (pRBC; 200 mL), aminocaproic acid^1^ (50 mg/kg IV q12h), yunnan baiyao^2^ (1 capsule PO q12h), and ondansetron^3^ (0.2 mg/kg PO q8h). After pRBC transfusion, peripheral PCV and TS were 29% and 6.8 g/dL, respectively.

The next day, prothrombin time (PT; 7.5 s; RI, 7.0–12.5 s), partial thromboplastin time (PTT; 12 s; RI, 8.5–15.5 s) and buccal mucosal bleeding time (BMBT; 3 min 30 s; RI, < 4 min) were measured and found to be within normal limits. Computed tomography of the abdomen identified concern for chronic hemorrhage at the site of the ovarian pedicles, prompting exploratory laparotomy. Intra‐operatively, the ovarian pedicles had robust clots bilaterally, and a Ligasure device (Covidien; Dublin, Ireland) was used to re‐seal surgical sites. Autotransfusion was performed during the exploratory laparotomy. Subcutaneous tissues were noted to be oozing during closure. Post‐operatively, PCV was stable with no further bleeding, and the dog was discharged 2 days later.

Two days after discharge (day 47 post‐ovariohysterectomy, day 4 post‐exploratory laparotomy), the dog was referred to the University of Wisconsin Veterinary Care for evaluation of persistent serosanguineous discharge from the laparotomy incision. Physical examination identified new ecchymoses in the inguinal region. A CBC identified regenerative anemia (Hct, 29%; RI, 39%–57%; reticulocytes, 0.113 × 10^6^/μL; RI, 0.013–0.102 × 10^6^/μL). Fibrinogen (263 mg/dL; RI, 69–289 mg/dL) and antithrombin (127%; RI, 71%–152%) were within normal limits but D‐Dimers were increased (410 ng/mL; RI, 0–135 ng/mL). Citrated‐kaolin thromboelastography (TEG) was performed and was consistent with hyperfibrinolysis (angle, 75.8°; RI, 34°–74°; lysis at 30 min [LY30], 12.9%; RI, 0%–5%). A hemophilia panel and factor XIII deficiency screening test were submitted to Cornell University College of Veterinary Medicine's Animal Health Diagnostic Center and did not identify any abnormalities. The remainder of the coagulation profile results were normal.

The archival DNA samples that had been submitted to the Comparative Coagulation laboratory from 2010 to 2020 based on breed designation (“Springer Spaniel”) and clinical histories of delayed post‐operative or post‐traumatic hemorrhage, absence of hemostasis test abnormalities, and response to empirical anti‐fibrinolytic drug treatment subsequently were recruited from the Cornell Veterinary Biobank.

The first case (Cornell ESS 1) was a 9‐month‐old, male castrated ESS diagnosed with hemoperitoneum after a fall, and a prior history of delayed hemorrhage and bruising after castration. Hemoperitoneum resolved after a packed red blood cell transfusion and IV aminocaproic acid that was maintained as PO treatment for 1 week. Hemostasis testing included platelet count, coagulation panel (activated partial thromboplastin time [APTT], PT, thrombin clotting time [TCT]), fibrinogen, platelet function analyzer‐100 (PFA100) closure time, and tissue factor and citrate‐native thromboelastography. All test results were within normal limits. The dog had no additional hemorrhagic events until 8 years of age when it was euthanized because of hemoperitoneum caused by rupture of a splenic mass.

The second case (Cornell ESS 2) was a 7‐month‐old, female spayed ESS with delayed and prolonged hemorrhage after ovariohysterectomy. Pre‐operative platelet count and coagulation panel (APTT, PT) were normal. Mild hemoperitoneum, first noted at 6 days after ovariohysterectomy, progressed over the course of 1 week and prompted exploratory laparotomy after pre‐operative stabilization with transfusions of fresh frozen plasma and packed red blood cells. Post‐operatively, the dog received PO aminocaproic acid and additional fresh frozen plasma transfusions. Peritoneal hemorrhage ultimately resolved after two recurrences with medical management consisting of an additional frozen plasma transfusion, PO tranexamic acid, and desmopressin acetate (intra‐ocular drops). Results of a coagulation panel (APTT, PT) submitted at the initial presentation for hemoperitoneum were normal, but plasma fibrinogen concentration was low (72 mg/dL; RI, 150–490 mg/dL). Subsequent hemostasis testing at the second recurrence of the hemoperitoneum indicated normal plasma fibrinogen concentration (159 mg/dL), normal coagulation panel results (APTT, PT, TCT) and normal antithrombin activity, Factor VIII and IX activities, and von Willebrand factor concentration. A third submission after complete resolution of bleeding and cessation of treatment identified normal plasma fibrinogen concentration (186 mg/dL), normal clot stability in a Factor XIII deficiency screening test [14], normal expression of platelet fibrinogen receptor (GpIIbIIIa complex) and normal platelet procoagulant activity in a flow cytometric assay [16]. The dog has had no additional bleeding events or other signs of illness through 5 years of observation since initial presentation.

The third case (Cornell ESS 3) was an 8‐month‐old male castrated ESS with recurrent hemorrhage after castration for cryptorchidism. The dog was re‐examined 24 h post‐operatively for hemorrhage at the inguinal incision site and was managed with wound debridement, resuturing, and packed red blood cell transfusion. Surgical site bleeding recurred approximately 12 h later. Hemorrhage resolved and did not recur after transfusion with fresh whole blood, fresh frozen plasma, and a 24‐h infusion of aminocaproic acid. Pre‐operative platelet count, APTT, PT, and BMBT were normal. Ancillary testing 1 month after initial presentation identified normal clot stability in a Factor XIII deficiency screening test [14], normal expression of platelet fibrinogen receptor (GpIIbIIIa complex), and normal platelet procoagulant activity in a flow cytometric assay [16].

The fourth case (Cornell WSS 1) was a 4‐year‐old female spayed WSS with delayed and recurrent hemoperitoneum after ovariohysterectomy. The dog had an exploratory laparotomy, and all surgical ligatures were noted to be intact. Perioperatively, the dog received multiple transfusions of fresh whole blood and fresh frozen plasma with a transient response. Ultimately, the dog was stabilized with no further hemorrhage at approximately 4 weeks after the initial presentation while receiving aminocaproic acid. Pre‐operative platelet count and coagulation panel (APTT, PT) were within normal limits. Ancillary testing identified no abnormalities in the coagulation panel (APTT, PT, TCT), procoagulant factor activities (Factors VIII, IX, XI), von Willebrand factor concentration, plasminogen activity, or antiplasmin activity [14, 16]. The dog was lost to follow‐up, with no subsequent clinical history available.

### 3.2 Genetic Analyses

Whole genome sequencing identified 180,895 unique variant positions and 272,648 unique variant effects in the proband (Table S3). Of these unique variant effects, 158 were predicted to have high, 664 moderate, 899 low, and 270,927 modifier impacts. Among the identified candidate genes (Supplemental Table S1), the proband was homozygous for an insertion in exon 1 of *SERPINE1* (NM_001197095.1:c.101dup) at chr6:8640592 that is predicted to cause a frameshift mutation and result in a downstream premature stop codon (NP_001184024.1:p.Ala35GlyfsTer56) in the gene transcript. This variant was absent from all dogs in the WGS database released by the Dog10K consortium. *SERPINE1* encodes for PAI‐1, which is a major inhibitor of fibrinolysis. No high‐ or moderate‐impact variants unique to the proband were identified in any of the other 11 candidate genes. Mapped alignments of the 12 candidate genes were scanned visually to search for structural variants, but none were identified. The remaining 157 variant effects with predicted high impact and 664 variant effects with predicted moderate impact were manually reviewed and were not found to be located in genes canonically associated with the fibrinolytic pathway.

**FIGURE 1:**
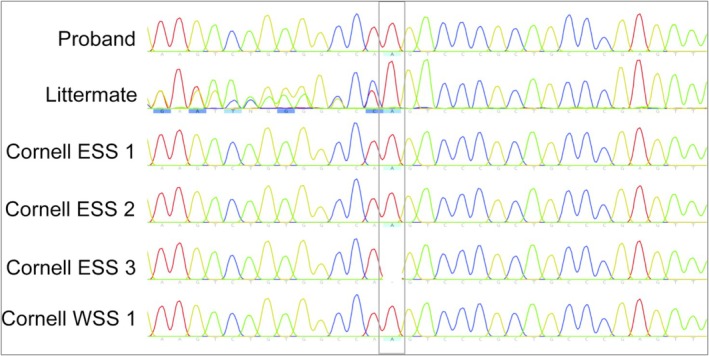
Sanger sequencing chromatograms surrounding the reported insertion in exon 1 of the *SERPINE1* gene at position chr6:8640592 (gray box) for the proband, her littermate, and three English Springer Spaniels (ESS) and one Welsh Springer Spaniel (WSS) from the Cornell University Veterinary Biobank with a history of unexplained hemorrhage. A homozygous insertion is present in the proband and three of the four dogs from Cornell. The proband's littermate is heterozygous for the same insertion, and one of the four dogs from Cornell (ESS 3) is homozygous for the reference allele.

Sanger sequencing of the proband's healthy littermate showed that it was heterozygous for the described *SERPINE1* mutation. Two ESS (ESS 1 and ESS 2) and the WSS from Cornell University with unexplained hemorrhage were homozygous for the same mutation, but the fourth dog from Cornell University (ESS 3) was homozygous for the normal reference gene (Figure 1). Among the 196 ESS with no known history of hemorrhage from the University of Minnesota (UMN) archives, one dog was heterozygous for the *SERPINE1* mutation. The remaining 195 dogs from UMN and the three additional ESS from the University of Wisconsin, of which platelets were evaluated for PAI‐1, were homozygous for the reference allele.

### 3.3 Liquid Chromatography–Tandem Mass Spectrometry

Across the six platelet samples submitted for LC–MS/MS, 188 proteins were detected with high confidence. Among these proteins, only peptides derived from PAI‐1 (UniProt ID: A0A8I3MDS4) were entirely absent in the proband (in both replicates) and present in the other four dogs, including the proband's littermate that was heterozygous for the identified *SERPINE1* mutation. Eight unique peptides were detected from PAI‐1 in these four animals, representing 24% amino acid coverage (TableS4) with 110–118 peptide spectrum matches per animal.

## 4 Discussion

We describe here a novel mutation in the *SERPINE1* gene in an ESS with hyperfibrinolysis and secondary hemoperitoneum. The duplication of an adenine base in exon 1 of the *SERPINE1* gene is predicted to cause a downstream frameshift mutation that encodes for a premature stop codon leading to absence of the PAI‐1 protein. This mutation is the first report of PAI‐1 deficiency causing hyperfibrinolysis and clinical hemorrhage in veterinary medicine and of primary congenital hyperfibrinolysis in a non‐sighthound breed.

Clinically, DEPOH secondary to hyperfibrinolysis has been described in Greyhounds with a relative deficiency of antiplasmin activity [14] and in association with a *SERPINF2* nucleotide variant in Scottish Deerhounds [15]. In both breeds, DEPOH and hyperfibrinolysis have been associated with abnormalities in alpha‐2 antiplasmin, a major circulating inhibitor of plasmin‐mediated fibrinolysis [14, 15]. Alpha‐2 antiplasmin binds directly to plasmin or fibrin to inhibit clot breakdown, and therefore loss of this inhibition leads to increased fibrinolysis. In contrast to these studies, our study detected a mutation in the *SERPINE1* gene that is associated with absent platelet PAI‐1 in an ESS. Homozygosity for the same mutation also was found in two unrelated ESS and in a WSS with delayed and prolonged post‐operative hemorrhage, supporting a pathologic role for this mutation in these two related breeds. Plasminogen activator inhibitor‐1 is a serine protease inhibitor (serpin) that regulates fibrinolysis by directly binding to and inhibiting tPA and uPA, both of which activate plasminogen to plasmin [32]. Plasmin mediates fibrin breakdown, and loss of inhibition of plasminogen conversion to plasmin leads to dysregulated and premature clot breakdown. Lack of PAI‐1 or alpha‐2 antiplasmin inhibitory actions on fibrinolysis might result in dysregulated or excessive fibrinolysis with resultant signs of a clinical bleeding disorder [33].

The laboratory diagnosis of hyperfibrinolysis is challenging because of the lack of gold standard global assays or sensitive screening tests for routine clinical use. In addition to the inherent complexity of the fibrinolytic system, species differences require adaptation and optimization of assays used in humans to evaluate fibrinolysis in dogs. Whole blood viscoelastic assays incorporate the contribution of cellular and fluid phase reactants for dynamic readouts of clot formation and lysis. However, these assays are nonspecific tests of the fibrinolytic pathway because of the influence of cell count, plasma fibrinogen concentration, and the procoagulant, thrombin‐generating potential of the test sample. Whole blood assays require on‐site blood collection for immediate analysis and dedicated viscoelastic monitors, thus restricting their availability. Viscoelastic testing was performed on two of the ESS examined at academic veterinary hospitals, with evidence of hyperfibrinolysis only found in the proband. Transfusion of packed red cells 24 h before testing might have influenced results for the other ESS. The addition of exogenous tPA has been reported to identify differences in fibrinolysis in viscoelastic assays that are not apparent in native samples [34]. Additional studies will be required to determine whether tPA‐modified viscoelastic assays can identify enhanced fibrinolysis associated with the *SERPINE1* variant in ESS.

Two genetic defects leading to PAI‐1 deficiency have been described in humans [35]. One defect is similar to our findings in which a frameshift mutation within exon 4 leads to a premature stop codon and is predicted to result in a truncated, non‐functional protein [4, 35]. The other defect is a non‐synonymous polymorphism that leads to a defective PAI‐1 protein [35]. Plasminogen activator inhibitor‐1 deficiency causing primary congenital hyperfibrinolysis in humans is linked with post‐operative and post‐traumatic hemorrhage, as well as severe menstrual bleeding [4, 5, 6, 7, 35]. Human patients with congenital PAI‐1 deficiency have clinical bleeding tendencies if they are homozygous for either mutation [35]. Heterozygous human patients generally are asymptomatic [6], but a case report describes a heterozygous individual who had spontaneous hemoarthrosis [36].

In our study, we genotyped 4 ESS (the proband and 3 banked DNA samples) and 1 WSS with post‐operative hemorrhage of unknown cause, and unaffected dogs including the proband's healthy littermate, three additional healthy ESS, and 196 banked ESS DNA samples from an unrelated study. Of the five clinically affected dogs, four were homozygous for the novel mutation and one ESS lacked the mutation. The proband's littermate was heterozygous for this mutation and has not had any reported clinical signs of hemorrhage after neutering or tail docking. This outcome is supported by the presence of PAI‐1 detected in the littermate's platelets. However, current testing of PAI‐1 concentrations or function in dogs is limited, including no measurement of plasma PAI‐1 concentration or activity in dogs heterozygous or homozygous for the *SERPINE1* mutation. It is possible that PAI‐1 detection in platelets might not be predictive of bleeding risk. We also found one dog among the 196 banked ESS DNA samples that was heterozygous for the reported mutation, suggesting that this genetic variant is present at a low frequency in the broader ESS population. The clinically affected WSS was homozygous for the *SERPINE1* mutation, suggesting that this variant might predate recognition of WSS as a separate breed in the early 1900s [37]. This finding is consistent with an autosomal recessive inheritance pattern and sporadic cases in which two heterozygous individuals are bred together and some offspring are affected. Clinical signs in the affected ESS and WSS were consistent with previously described case reports of delayed and prolonged post‐operative hemorrhage, severe subcutaneous bleeding, and cavitary hemorrhage in humans with PAI‐1 deficiency. Additional studies of these related breeds are indicated to better understand the frequency of this *SERPINE1* mutation and its associated clinical phenotypes.

In conclusion, homozygosity for a novel *SERPINE1* mutation found in ESS and WSS was associated with a severe bleeding disorder characterized by delayed and prolonged post‐operative hemorrhage. Viscoelastic testing of whole blood samples from the proband identified hyperfibrinolysis, and proteomic analyses of the proband's platelets did not detect PAI‐1 protein. These results, and the similar clinical signs of *SERPINE1* mutations in people, indicate that this novel mutation is a likely causal disease gene. Future studies will be needed to further confirm and characterize the trait, including measurement of plasma and platelet PAI‐1 expression, and pedigree studies in ESS and WSS to establish a mode of inheritance and disease gene prevalence.

## Disclosure

Authors declare no off‐label use of antimicrobials.

## Ethics Statement

Approved by the University of Wisconsin Institutional Animal Care and Use Committee (number V006844). Authors declare human ethics approval was not needed.

## Conflicts of Interest

Steven G. Friedenberg serves as Associate Editor for the Journal of Veterinary Internal Medicine. He was not involved in the review of this manuscript. The other authors declare no conflicts of interest.

## Supporting information


**Table S1.** Candidate genes involved in the fibrinolytic pathway [19, 20, 21, 22, 23, 24, 25] that were searched for deleterious genetic variants as part of the study.

**Table S2.** Plasminogen activator inhibitor 1 (PAI‐1) peptide precursor values used in LC–MS/MS.

**Table S3.** List of unique variants in the proband using WGS.

**Table S4.** Eight unique peptides derived from PAI‐1 detected in the proband’s littermate and three English Springer Spaniels (ESS) without hemorrhage using LC–MS/MS.

## Endnotes

^1^
Pfizer; New York, NY, USA.

^2^
Yunnan Baiyao Group; Yunnan, China.

^3^
Glenmark Pharmaceuticals Inc.; Mahwah, NJ, USA.
