# Supplementary material for: Identification of a Novel Mutation in the SERPINE1 Gene Causing Clinical Hyperfibrinolysis in English Springer Spaniel Dogs
Source: J Vet Intern Med. 2025 Jun 5;39(4):e70150. doi: 10.1111/jvim.70150 (PMC12138437; doi:10.1111/jvim.70150)
Supplement: Supplementary file 1 — Table S1. Candidate genes involved in the fibrinolytic pathway [19, 20, 21, 22, 23, 24, 25] that were searched for deleterious genetic variants as part of the study. [file JVIM-39-e70150-s002.docx]

| Gene | Protein |
| --- | --- |
| CPB2^20^ | carboxypeptidase B2 |
| FGA^21^ | fibrinogen alpha chain |
| FGB^21^ | fibrinogen beta chain |
| FGG^21^ | fibrinogen gamma chain |
| PLAT^22^ | plasminogen activator, tissue type |
| PLAU^23^ | plasminogen activator, urokinase |
| PLG^24^ | plasminogen |
| SERPINA5^25^ | plasminogen activator inhibitor-3 |
| SERPINB2^25^ | plasminogen activator inhibitor-2 |
| SERPINE1^25^ | plasminogen activator inhibitor-1 |
| SERPINE2^25^ | peptidase inhibitor-7 |
| SERPINF2^25^ | alpha-2-antiplasmin |
