# Supplementary material for: Identification of a Novel Mutation in the SERPINE1 Gene Causing Clinical Hyperfibrinolysis in English Springer Spaniel Dogs
Source: J Vet Intern Med. 2025 Jun 5;39(4):e70150. doi: 10.1111/jvim.70150 (PMC12138437; doi:10.1111/jvim.70150)
Supplement: Supplementary file 2 — Table S2. Plasminogen activator inhibitor 1 (PAI‐1) peptide precursor values used in LC–MS/MS. [file JVIM-39-e70150-s003.docx]

| **Peptide Sequence** | **Precursor *m/z*** | **Precursor charge** |
| --- | --- | --- |
| QIQEAMQFQIDEK | 804.3903 | 2 |
| GAVDQLTR | 430.2352 | 2 |
| GMIGNLLGR | 465.7631 | 2 |
| FIVNDWVK | 510.7793 | 2 |
| AAGLATDFGVK | 525.2849 | 2 |
| SDGSTVSVPMMAQTNK | 826.8846 | 2 |
| GMIGNLLGR | 465.7631 | 2 |
| FSLETEVNLR | 604.3195 | 2 |
| QIQEAMQFQIDEK | 536.5960 | 3 |
| SDGSTVSVPMMAQTNK | 551.5921 | 3 |
| FSLETEVNLR | 403.2154 | 3 |
| ELMGPWN(deamidated)KDEISTADAIFVQR | 807.7300 | 3 |
