# Supplementary material for: Identification of a Novel Mutation in the SERPINE1 Gene Causing Clinical Hyperfibrinolysis in English Springer Spaniel Dogs
Source: J Vet Intern Med. 2025 Jun 5;39(4):e70150. doi: 10.1111/jvim.70150 (PMC12138437; doi:10.1111/jvim.70150)
Supplement: Supplementary file 4 — Table S4. Eight unique peptides derived from PAI‐1 detected in the proband’s littermate and three English Springer Spaniels (ESS) without hemorrhage using LC–MS/MS. [file JVIM-39-e70150-s004.docx]

| QIQEAMQFQIDEK |
| --- |
| GAVDQLTR |
| AAGLATDFGVK |
| ELMGPWNKDEISTADAIFVQR |
| FSLETEVNLR |
| SDGSTVSVPMMAQTNK |
| FIVNDWVK |
| GMIGNLLGR |
